# Tuberculous brain abscess mimicking metastatic disease

**DOI:** 10.1007/s15010-026-02916-6

**Published:** 2026-08-11

**Authors:** Anson Chung-Ho Lee, Wing-Sze Tong

**Affiliations:** 1 State Key Laboratory of Emerging Infectious Diseases, Carol Yu Centre for Infection, Department of Microbiology, School of Clinical Medicine, Li Ka Shing Faculty of Medicine, The University of Hong Kong, Pokfulam, Hong Kong SAR China; 2 Department of Pathology, Kwong Wah Hospital, Kowloon, Hong Kong SAR China

**Keywords:** Brain abscess, Tuberculosis, *Mycobacterium tuberculosis*, Acid-fast bacilli, Central nervous system infection

## Abstract

An 85-year-old, HIV-seronegative woman presented with subacute right-sided weakness and dysarthria. Neuroimaging revealed multiple rim-enhancing intracranial mass lesions, initially attributed to metastatic disease of unknown primary. Subsequent biopsy and excision yielded purulent material with abundant acid-fast bacilli; culture and molecular testing confirmed *Mycobacterium tuberculosis*. Histopathology demonstrated an exceptionally high mycobacterial burden and an absence of well-formed epithelioid granulomas, establishing a diagnosis of tuberculous brain abscess. A 12-month anti-tuberculosis therapy led to clinical and radiological resolution. This case illustrates an uncommon presentation of central nervous system tuberculosis that can closely mimic intracranial malignancy.

An 85-year-old woman presented with 1 month of right-sided weakness and 1 week of dysarthria, without fever or headache. Examination revealed cachexia and right hemiparesis without meningism.

Gadolinium-enhanced brain MRI demonstrated nodular meningeal thickening along the left cerebral convexity, multiple rim-enhancing cystic masses in the left frontal and parietal lobes, perilesional oedema, and mild rightward midline shift (Fig. 1). Whole-body FDG PET-CT showed no primary tumour.

Metastatic disease of unknown primary was suspected. The patient was initially referred for whole-brain radiotherapy, which was deferred for a biopsy of the dominant left parietal lesion. This yielded purulent material containing abundant acid-fast bacilli (AFB); culture and molecular testing confirmed *Mycobacterium tuberculosis*; histology was negative for malignancy.

Surgical excision of the dominant abscess revealed a thick capsule containing viscous pus (Fig. 2). Histopathology showed extensive necrosis and vascularised granulation tissue with mixed, predominantly neutrophilic infiltrates (Fig. 3). Ziehl-Neelsen staining revealed dense clusters of AFB (Fig. 4). The patient was HIV-seronegative. Following 12 months of antituberculosis therapy (initiated with isoniazid, rifampicin, pyrazinamide and levofloxacin) and an 8-week tapering course of dexamethasone (starting at 0.4 mg/kg/day IV, transitioning to oral), imaging demonstrated complete resolution of the abscesses, with clinical stability 2 months post-treatment.

Unlike typical paucibacillary tuberculomas, tuberculous brain abscess (TBA) is characterised by a neutrophil-predominant infiltrate, an exceptionally high bacillary load, and an absence of well-formed epithelioid granulomas [1, 2]. These features reflect impaired cell-mediated immunity, likely attributable to advanced age and poor nutritional state in this patient [3, 4]. Clinicians should consider TBA in the differential diagnosis of intracranial mass lesions to avoid diagnostic delay.

**Fig. 1:**
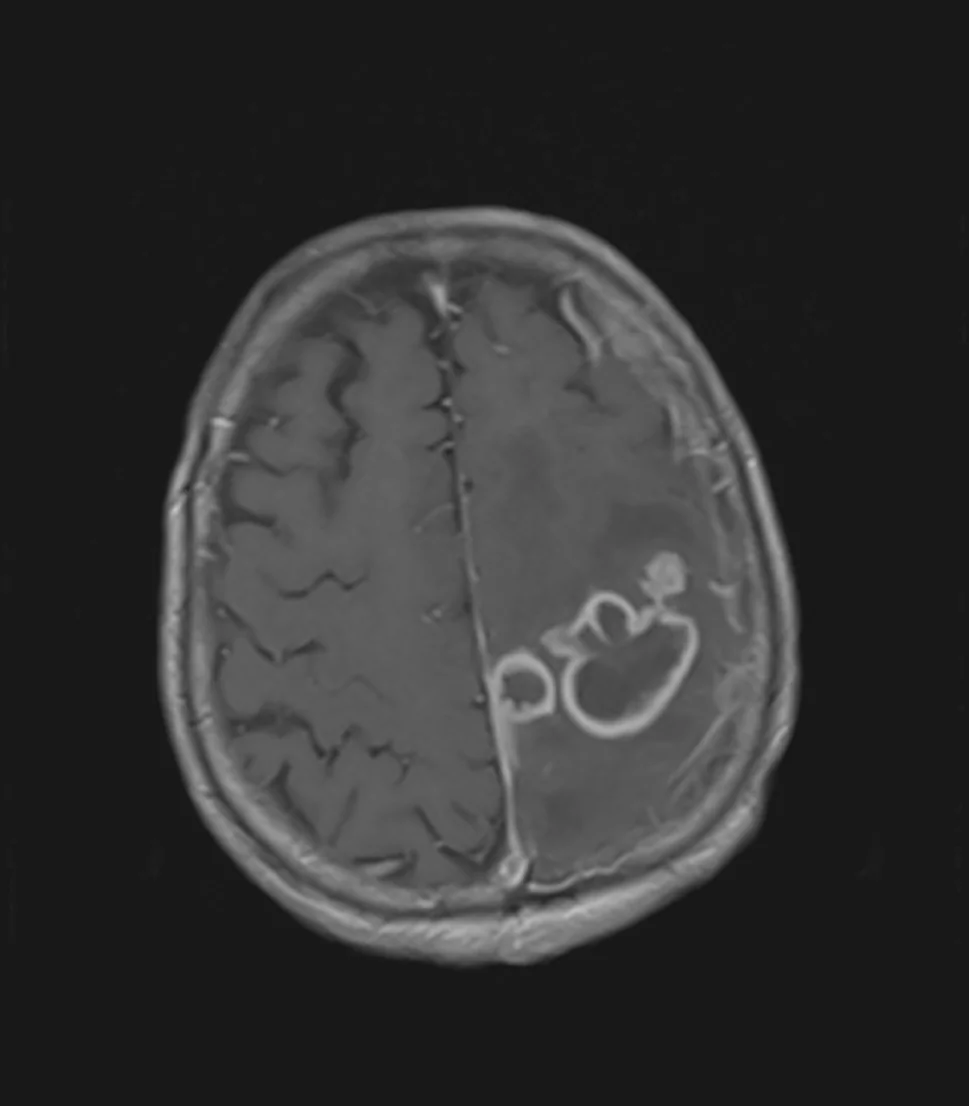
Gadolinium-enhanced MRI showing rim-enhancing cystic masses in the left frontal and parietal lobes with T1 hypointense content, prominent perilesional oedema, and mild rightward midline shift

**Fig. 2:**
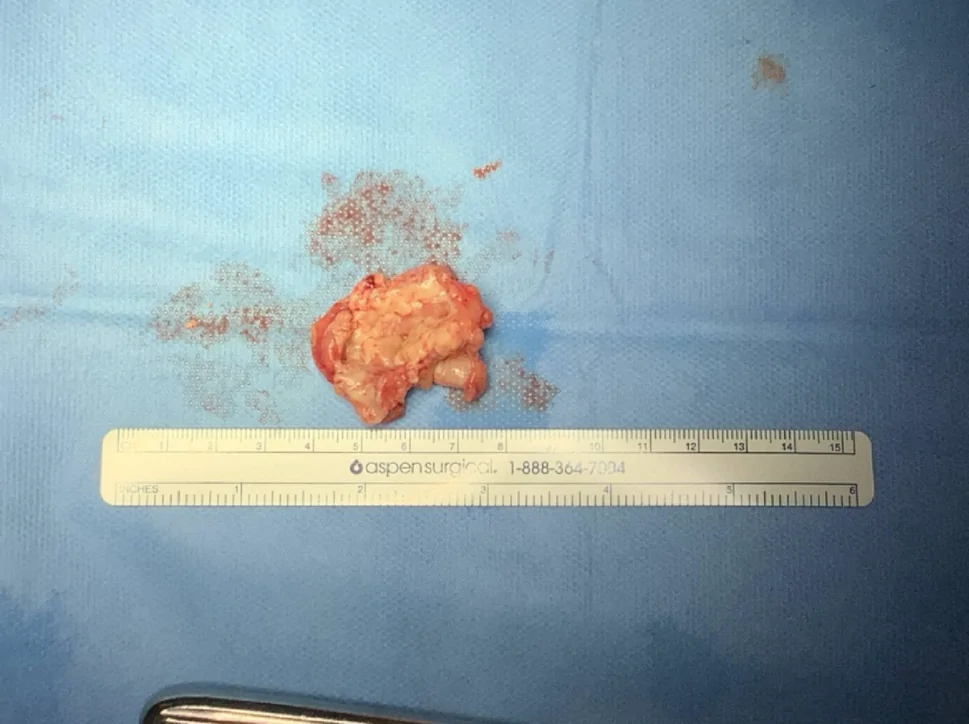
Resected dominant abscess with a thick capsule containing viscous pus

**Fig. 3:**
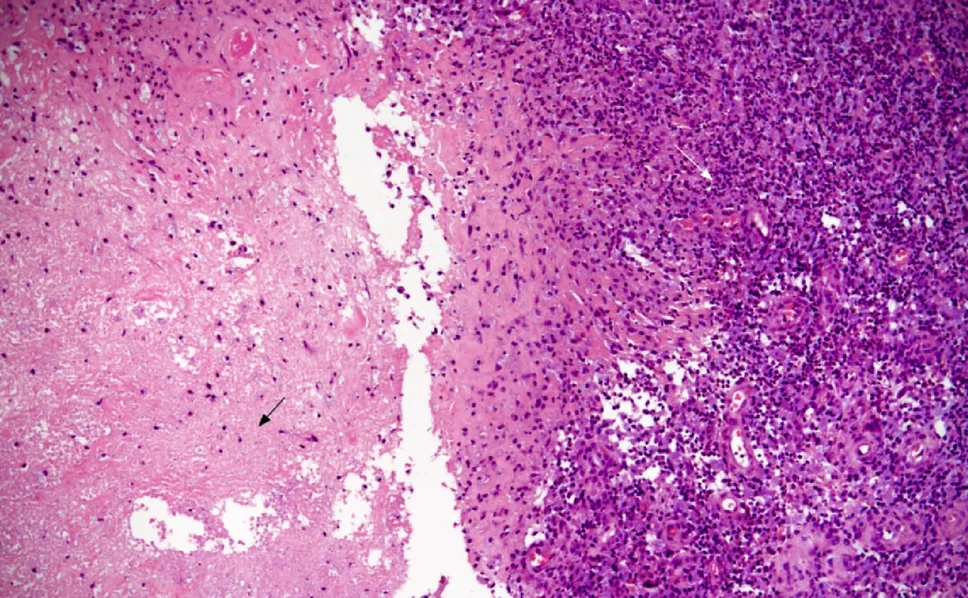
Histopathology of the abscess wall showing extensive necrosis (black arrow) and vascularised granulation tissue with mixed, predominantly neutrophilic, inflammatory infiltrates (white arrow)

**Fig. 4:**
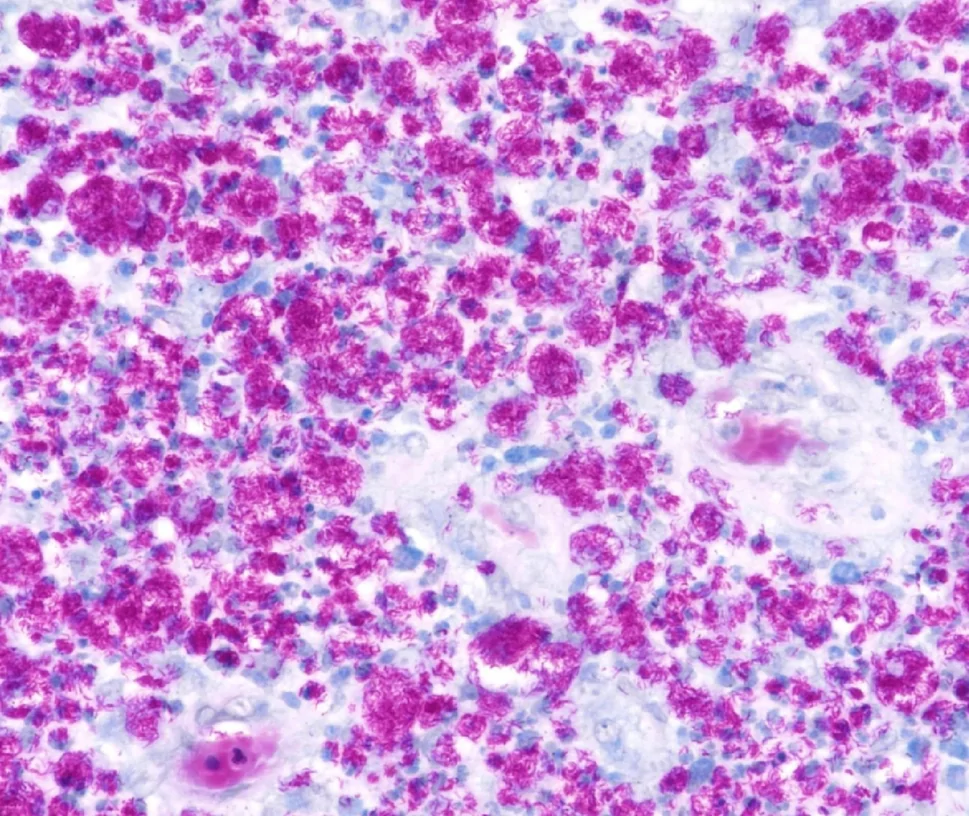
Ziehl-Neelsen staining of the abscess showing dense clusters of AFB

## Data Availability

No datasets were generated or analysed during the current study.
